# Excerpta

**Published:** 1880-06

**Authors:** 


Salicylate of Potash.—This salt is very strongly recommended Rheumatism by Dr. Donnelly in the *N. Y. Medical Record.* His prescription is as follows :—

℞.—Acid Salycilic................................ ʒ iii

Potass. Bicarbon.......................... ʒ vi.

Aquæ.................................. ....... ℥ ii.

Sig. A teaspoonful every three hours. He says :—"The rapidity of action of this combination is remarkable. Its absorption seems to be immediate; the patient will speak of the speedy relief he experiences; the blood is restored to its natural alkalinity, as seen by the diminished acidity of the perspiration and urine; the brick-dust sediment in the latter disappears, the pain and swelling soon subside, and the metastatic character of the disease is lost. Judging from the effects in a dozen cases, it is no over-estimate to say that salicylate of potash is as thoroughly abortive of acute rheumatism as quinine is of intermittent fever." It is quite possible that this alkaline salt has some advantages over the soda salt which has been so commonly employed, and at any rate is well worthy of a trial. *From Canada Medical and Surgical Journal.*

United States Postal Law.—1. A postmaster is required to give notice *by letter* (returning a paper does not answer the law) when a subscriber does not take his paper out of the office, and state the reason for its not being taken. Any neglect to do so makes the postmaster *responsible* to the publishers for payment.

2. Any person who takes a paper from the post-office, whether directed to his name or another, or whether he has subscribed or not, is responsible for the pay.

3. If a person orders his paper discontinued, he must pay all arrearages, or the publisher may continue to send it until payment is made, and collect the whole amount, *whether it be taken from the office or not.* There can be no legal discontinuance until the payment is made.

4. If the subscriber orders his paper to be stopped at a certain time, and the publishers continues to send, the subscriber is bound to pay for it *if he takes it out of the post-office.* The law proceeds upon the ground that a man must pay for what he uses.

5. The courts have decided that refusing to take a newspaper and periodicals from the post-office, or removing and leaving them uncalled for, is *prima facia* evidence of intentional fraud.

